# Supplementary material for: Pulsed Sagnac source of polarization-entangled photon pairs in telecommunication band
Source: Sci Rep. 2019 Mar 22;9:5031. doi: 10.1038/s41598-019-41633-z (PMC6430775; doi:10.1038/s41598-019-41633-z)
Supplement: Supplementary file 1 — Pulsed Sagnac source of polarization-entangled photon pairs in telecommunication band [file 41598_2019_41633_MOESM1_ESM.pdf]

Supplementary information:

Pulsed Sagnac source of polarization-entangled photon pairs in telecommunication band

Heonoh Kim,[1] Osung Kwon,[2,†] Han Seb Moon[1,*]
[1]Department of Physics, Pusan National University, Geumjeong-Gu, Busan 46241, South Korea
[2]National Security Research Institute, Daejeon 34044, South Korea
[†]E-mail: oskwon@nsr.re.kr
[*]Corresponding author: hsmoon@pusan.ac.kr

Supplementary Figures and Table

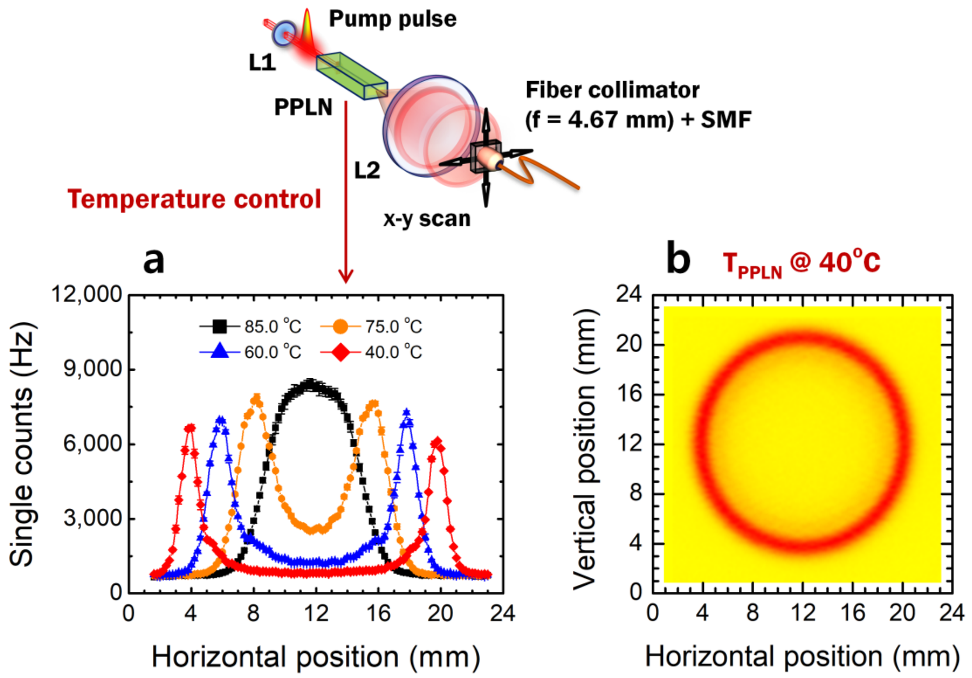

Figure S1. Imaging of non-collinear QPM-SPDC spectra. Correlated photon pairs in the 1550-nm telecommunication band are generated via non-collinear QPM-SPDC in a 10-mm-long type-0 PPLN crystal. L1 and L2 are spherical lenses with 200-mm focal length and diameters of 1 and 2 inch, respectively. Spatially distributed emission spectra are collected by imaging optics positioned after collimating lens L2. A bandpass filter with 10-nm bandwidth is attached in front of the imaging optics, which consists of SMF-collimator employing an aspherical lens with a 4.67-mm focal length (Numerical aperture: 0.53) and a two-dimensional translation stage. (**a**) Measured single-photon counting rates as functions of coupling-optics horizontal position for various PPLN temperatures. In this measurement, the vertical position of the collimator is fixed at the center position (y = 12 mm). (**b**) Non-collinear emission spectra measured with the imaging optics behind L2 when the PPLN temperature (T_{PPLN}) was set to 40°C. The imaging-optics step size was 0.2 mm in both the horizontal and vertical positions.

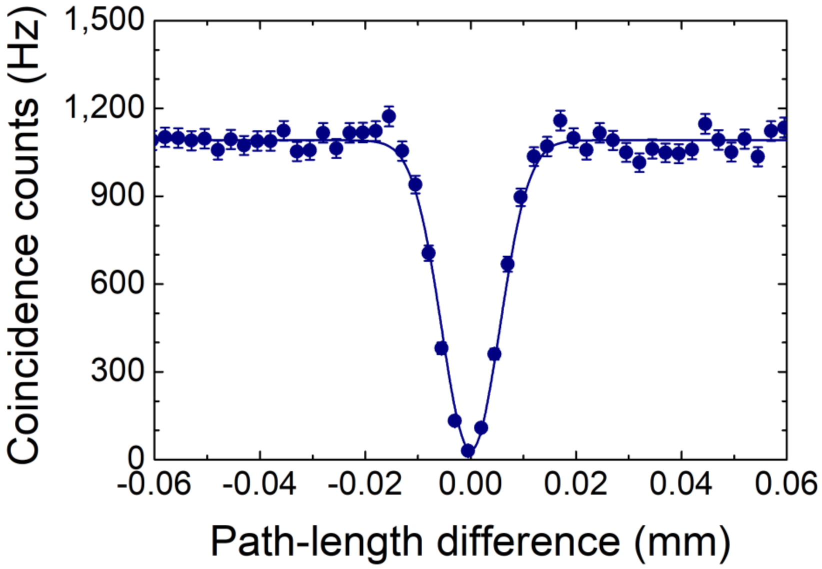

Figure S2. Hong-Ou-Mandel interference fringe measured without filters. Broadband spectra were emitted from the PPLN crystal under the condition of a non-collinear type-0 QPM SPDC. The SMF-coupled bandwidth of the generated photons was found to be approximately 132 nm, which was estimated from the Hong-Ou-Mandel interference-fringe width. The SMF coupling optics used in this measurement was also employed in the Sagnac source.

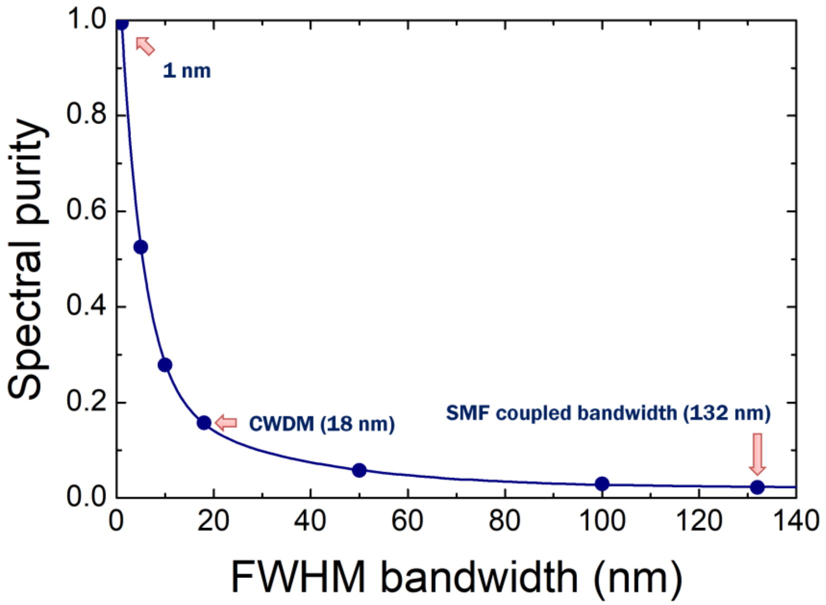

Figure S3. Spectral purity vs spectral bandwidth. The spectral purity of the SMF-coupled photon-pair source was estimated from the Schmidt numbers for given spectral bandwidths.

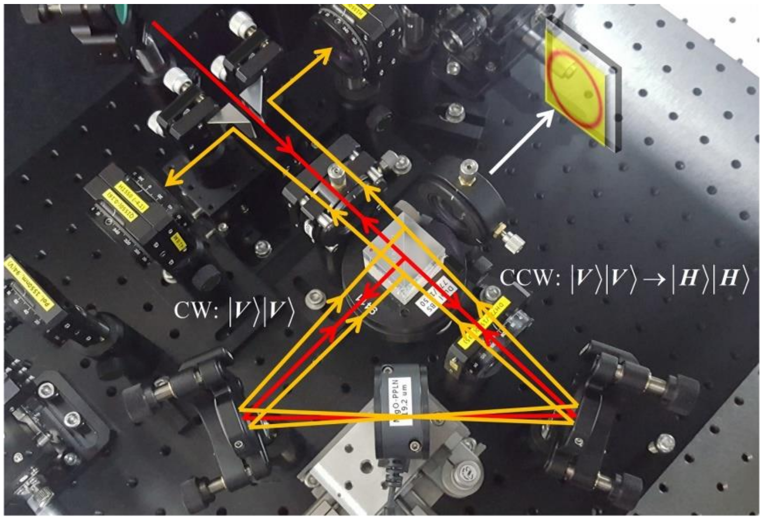

Figure S4. Photograph of polarization-Sagnac source. All optical components for source construction were positioned on 45 x 60 cm^2 breadboard. The Sagnac loop area was 10 x 10 cm^2.

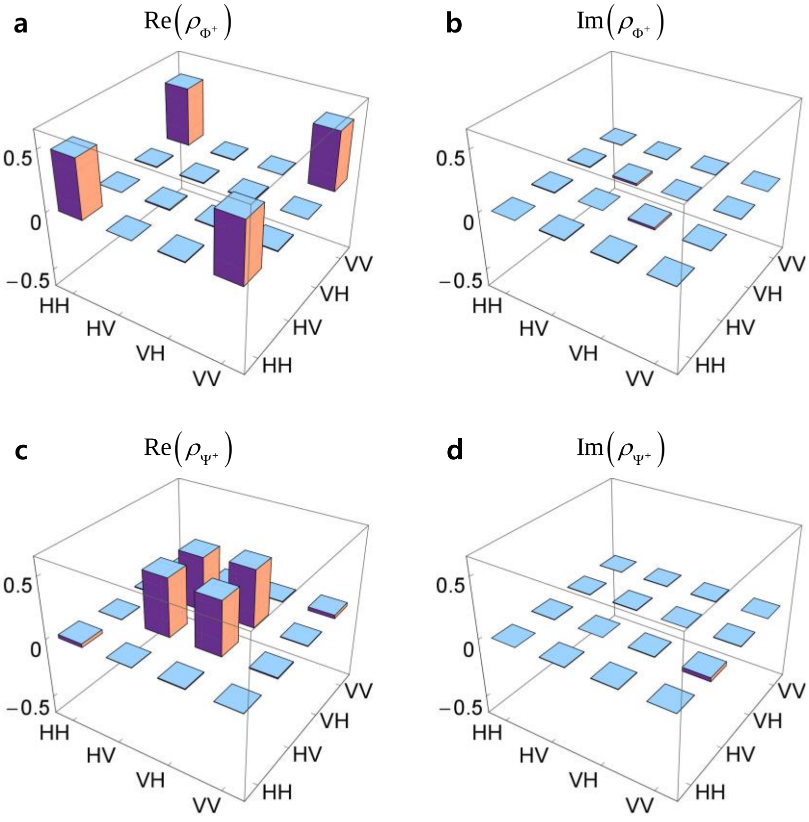

Figure S5. Reconstructed density matrices for $|\Phi^+\rangle$ and $|\Psi^+\rangle$ states. To verify the entanglement quality of the $|\Phi^+\rangle$ and $|\Psi^+\rangle$ states, we performed the quantum state tomography with coincidence events obtained from 16 measurement bases. (**a,c**) Real and (**b,d**) imaginary parts of the reconstructed density matrices. The fidelities of the reconstructed density matrices were 0.851 (0.965) for $|\Phi^+\rangle$ state and 0.834 (0.952) for $|\Psi^+\rangle$ state from the raw (net) coincidences, respectively.

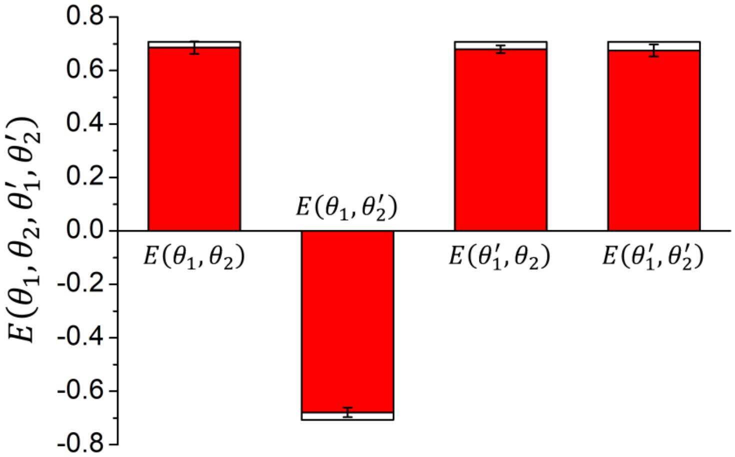

Figure S6. Polarization-correlation coefficients for $|\Phi^+\rangle$ state. To verify the experimental violation of the CHSH-Bell inequality for the $|\Phi^+\rangle$ state, we measured the correlation coefficients in Eq. (3) of the main text, when the angles of the two polarizers were set to $\theta_1 = 0^\circ$, $\theta_2 = 22.5^\circ$, $\theta_1' = 45^\circ$, and $\theta_2' = 67.5^\circ$. To obtain identical absolute heights for the histograms, the angle offsets of the two linear polarizers were carefully adjusted for the polarization-correlation measurements. The filled (blank) areas represent the measured (ideal) correlation coefficients and the error bars denote the standard deviations estimated from the measured coincidence counting uncertainties. The experimentally obtained correlation coefficients were $E(\theta_1, \theta_2)$ = 0.6857 ± 0.0229, $E(\theta_1, \theta_2')$ = -0.6797 ± 0.0179, $E(\theta_1', \theta_2)$ = 0.6795 ± 0.0148, and $E(\theta_1', \theta_2')$ = 0.6745 ± 0.0225, which yielded a CHSH-Bell parameter *S* value of 2.7194 ± 0.0396. Without subtraction of the accidental coincidences, the raw *S* value of the CHSH-Bell inequality was 2.4878 ± 0.0370.

Table 1. Comparison of the polarization-entangled photon-pair sources based on the quasi-phase matched (QPM) SPDC, which are implemented in the Sagnac configuration. *PGR, pair generation rate per pulse; *SNSPD, superconducting nanowire single-photon detector; *c-SHG/SPDC, cascaded SHG and the following SPDC.

SPDC type	Phase matching<br>Detector	Visibility/<br>Fidelity	Brightness<br>(Hz/mW/nm)	References
Type-II<br>Collinear QPM<br>PPKTP, 10 mm	405 nm (cw)<br>→ 810/810 nm<br>Si-APD	V = 96.8%	5 x 10^3	Kim et al.[1]
Type-II<br>Collinear QPM<br>PPKTP, 25 mm	405 nm (cw)<br>→ 810/810 nm<br>Si-APD	V = 99.5%	2.73 x 10^5	Fedrizzi et al.[2]
Type-0<br>Collinear QPM<br>PPLNWG, 1 mm	776 nm (300 fs, 73 MHz)<br>→ 1542/1562 nm<br>InGaAs-APD	F = 0.863	*PGR = 0.185	Lim et al.[3]
Type-0<br>Collinear QPM<br>PPLNWG, 10 mm	1548.6 nm (120 ps, 40 MHz)<br>→ 1538.8/1558.66 nm<br>*c-SHG/SPDC<br>InGaAs-APD	V = 96%<br>F = 0.97	*PGR = 0.04	Arahira et al.[4]
Type-II<br>Collinear QPM<br>PPKTP, 15 mm	404 nm (2 ps, 76 MHz)<br>→ 808/808 nm<br>Si-APD	V = 98.7%<br>F = 0.982	1.78 x 10^4	Predojević et al.[5]
Type-II<br>Collinear QPM<br>PPKTP, 30 mm	792 nm (2 ps, 76 MHz)<br>→ 1584/1584 nm<br>*SNSPD	V = 96%<br>F = 0.98	*PGR = 0.014	Jin et al.[6]
Type-II<br>Collinear QPM<br>PPKTP, 10 mm	775 nm (cw)<br>→ 1550/1550 nm<br>InGaAs-APD	V = 96.4%<br>F = 0.935	2	Li et al.[7]
Type-0<br>Non-collinear QPM<br>PPKTP, 30 mm	405 nm (cw)<br>→ 810/810 nm<br>Si-APD	V = 97%<br>F = 0.975	0.025	Jabir et al.[8]
Type-0<br>Non-collinear QPM<br>PPLN, 10 mm	775 nm (3.5 ps, 20 MHz)<br>→ 1550/1550 nm<br>InGaAs-APD	V = 96%<br>F = 0.965	*PGR = 0.046	Our work

References

1. Kim, T., Fiorentino, M. & Wong, F. N. C. Phase-stable source of polarization-entangled photons using a polarization Sagnac interferometer. *Phys. Rev. A* **73**, 012316 (2006).

2. Fedrizzi, A., Herbst, T., Poppe, A., Jennewein, T. & Zeilinger, A. A wavelength-tunable fiber-coupled source of narrowband entangled photons. *Opt. Express* **15**, 15377-15386 (2007).

3. Lim, H. C., Yoshizawa, A., Tsuchida, H. & Kikuchi, K. Stable source of high quality telecom-band polarization-entangled photon-pairs based on a single, pulse-pumped, short PPLN waveguide. *Opt. Express* **16**, 12460-12468 (2008).

4. Arahira, S., Kishimoto, T. & Murai, H. 1.5-μm band polarization entangled photon-pair source with variable Bell states. *Opt. Express* **20**, 9862-9875 (2012).

5. Predojević, A., Grabher, S. & Weihs, G. Pulsed Sagnac source of polarization entangled photon pairs. *Opt. Express* **20**, 25022-25029 (2012).

6. Jin, R.-B. *et al*. Pulsed Sagnac polarization-entangled photon source with a PPKTP crystal at telecom wavelength. *Opt. Express* **22**, 11498-11507 (2014).

7. Li, Y., Zhou, Z.-Y., Ding, D.-S. & Shi, B.-S. CW-pumped telecom band polarization entangled photon pair generation in a Sagnac interferometer. *Opt. Express* **23**, 28792-28800 (2015).

8. Jabir M. V. & Samanta, G. K. Robust, high brightness, degenerate entangled photon source at room temperature. *Sci. Rep*. **7**, 12613 (2017).
